# Identifying regulatory outcomes of Non-interventional Post-Authorisation Safety Studies (PASS) in the European repository of studies using publicly available information

**DOI:** 10.3389/fdsfr.2025.1574430

**Published:** 2025-09-10

**Authors:** M. F. Almas, A. Girardi, S. Crisafulli, K. Gvozdanovic, K. M. Hakkarainen, G. Hyeraci, W. E. Hoogendoorn, B. Rillmann, G. Roberto, G. Vitturi, G. Trifirò, ENCePP WG3

**Affiliations:** ^1^ IQVIA Epidemiology and Database Studies, Real World Solutions, Lisbon, Portugal; ^2^ Agenzia Regionale di Sanità Toscana, Florence, Italy; ^3^ Department of Diagnostics and Public Health, University of Verona, Italy; ^4^ Agency for Medicinal Products and Medical Devices, Zagreb, Croatia; ^5^ Andrija Stampar Teaching Institute of Public Health, Zagreb, Croatia; ^6^ Epidemiology and Real-World Science, Parexel, Gothenburg, Sweden; ^7^ Real World Solutions, IQVIA Epidemiology and Database Studies, Real World Solutions, Amsterdam, Netherlands; ^8^ Late Phase and Real World Evidence ICON Plc, Mannheim, Germany

**Keywords:** post-authorisation safety studies (PASS), regulatory outcomes, European Medicines Agency, transparency, risk management, EU PAS register

## Abstract

**Background:**

Post-Authorisation Safety Studies (PASS) generate evidence that may support regulators to evaluate the benefit-risk profile of medicinal products and to take regulatory actions when needed. The European Medicine Agency (EMA) promoted decision-making transparency by publishing regulatory guidelines and endorsing the registration of PASS in the European Post‐Authorisation Study (EU PAS) Register (currently HMA-EMA Catalogues of real-world studies). A recent analysis of the EU PAS Register showed a marked increase of PASS conducted as multidatabase studies (MDS) during the last decade.

**Purpose:**

This study aimed to assess the feasibility of using public available information from the EMA website to identify regulatory outcomes of PASS and, to describe the retrieved regulatory outcomes of PASS by MDS and non-MDS.

**Methods:**

Multidatabase PASS, and an equal number of non-multidatabase PASS, were selected from the EU PAS Register if: completed between 07/2012 and 12/2020, part of the Risk Management Plan (RMP) category 1,2 or 3, required by an European regulator, and conducted in ≥1 European country including United Kingdom (UK). Mentions of the identified PASS were searched in the Pharmacovigilance Risk Assessment Committee (PRAC) meeting minutes, the procedural steps document of the European Public Assessment Report (EPAR), and other publicly available EMA webpages, mainly using study title, acronym, protocol, or regulatory number. Whenever identified, the regulatory outcome information was retrieved.

**Results:**

A total of 84 PASS (42 MDS and 42 non-MDS) were included in the analysis. We could identify 77% of the 84 PASS in at least one of the used sources but the information available was scarce. Among the identified PASS, information on regulatory outcomes was identified for 60%, representing 46% of all 84 PASS, with no differences between MDS and non-MDS. Regulatory outcome information was more frequently identified for imposed (78%) than non-imposed PASS (34%). The availability of regulatory outcomes increased from 25% for PASS completed in the years 2012–2014 to 56% for those completed in 2018–2020. The regulatory outcome information was unspecific for two thirds of the PASS with available outcomes, limiting our description of regulatory outcome types.

**Conclusion:**

The attempt to identify and describe the regulatory outcomes of MDS registered into the EU PAS Register, and compare them to non-MDS, was impaired by the difficulties in retrieving the regulatory outcome insights from the publicly available sources such as, identifying PASS across sources, lack of information and granularity, standardisation and consistency across sources. We advocate for an increased transparency of the regulatory outcomes of PASS through the use of a unique PASS identifier for an easy link across sources, and for a complete regulatory outcome publication with enhanced and standardised presentation of regulatory outcomes to understand the regulatory impact of the PASS.

## Introduction

The European regulatory framework defines a Post-Authorisation Safety Study (PASS) as any study relating to an authorised medicinal product conducted with the aim of identifying, characterising, or quantifying a safety hazard, confirming the safety profile of the medicinal product, or measuring the effectiveness of risk management measures (The European Parliament and the Council of the European Union, 2022; European Medicines Agency, 2017). The 2012 Pharmacovigilance legislation set the legal basis for PASS which can be imposed as a condition to a marketing authorisation (i.e., Risk Management Plan (RMP) category 1 and 2 studies) or required in the RMP to investigate a safety concern or to evaluate the effectiveness of risk minimisation activities (i.e., RMP category 3) (The European Parliament and the Council of the European Union, 2022; European Medicines Agency, 2017). PASS enables the generation of potentially new evidence that competent authorities can leverage to take regulatory actions to support the safe and effective use of medicinal products. Such regulatory actions may include variations to the Marketing Authorisation (e.g., update of the summary of product characteristics [SmPC]), or even, the suspension, or revocation of the marketing authorisation (European Medicines Agency, 2017).

In addition to providing a legal framework for PASS, the 2012 legislation aimed at promoting transparency of the process of generation of post-marketing evidence and its use for regulatory purposes. This included the request to register PASS and publish study protocols and results in the European post-authorisation study (EU PAS) register, recently replaced by the “HMA-EMA Catalogue of real-world studies” (European Medicines Agency, 2025). Transparency is also promoted by the publication of regulatory decisions as part of the European public assessment report (EPAR) applicable to active substances contained in central authorisation procedures (CAP), a dedicated webpage for outcomes of imposed PASS for active substances contained in national authorisation procedures (NAP) or via the Pharmacovigilance Risk Assessment Committee (PRAC) meeting minutes, which are publicly accessible from the EMA website (Arlett et al., 2014; Kurz et al., 2018; European Network of Centres for Pharmacoepidemiology and Pharmacovigilance, 2023; European Medicines Agency, 2023b).

The EU PAS Register and PRAC meeting minutes have been previously used as sources to describe the PASS characteristics (Engel and Almas et al., 2017). A recent publication provided an overview of all Post-Authorisation Studies (PASS and other) performed in Europe and registered in the EU PAS Register between September 2010 and December 2018. Sultana et al. reported that almost two-third of all registered studies were observational based on secondary use of electronic healthcare data and about half of them were using multiple data sources (Sultana et al., 2022). However, regulatory outcomes were not investigated. Notably, the authors classified the studies into multidatabase studies (MDS) according to the available information in the EU PAS register (Sultana et al., 2022). Another publication did investigate regulatory outcomes but focused on PASS assessing the effectiveness of risk minimisation measures (RMM) and used non-public EMA sources (Grupstra et al., 2023). In recent years, the interest in MDSs has grown due to their potential to analyze healthcare data from heterogeneous sources and to gather a large number of patients, being particularly useful to study rare diseases/exposure and special populations. Currently, several database networks globally support informed regulatory decision making (e.g., Sentinel in the United States, DARWIN and VAC4EU (for vaccines) in the-EU, Cnodes in Canada, Italian MD network, etc.) (Food and Drug Administration, 2024; European Medicines Agency, 2024; CNODES, 2024; VAC4EU, 2019; Trifirò et al., 2021). Thus, the regulatory impact of MDS PASS is of interest. To the best of our knowledge, no published study has used publicly available regulatory documents to link non-interventional PASS registered in the EU PAS Register with corresponding regulatory outcomes and to find out whether these differ between MDS and non-MDS.

The aim of our study was twofold. On the one hand, to assess the feasibility of using publicly available information from the EMA website to identify PASS regulatory outcomes. On the other hand, to investigate and describe the retrieved regulatory outcomes of PASS by MDS and non-MDS.

## Materials and methods

### Study selection

The extract of the EU PAS Register from Sultana et al. which included any post-authorisation study since the start of EU PAS Register until December 2018 (Sultana et al., 2022), was used as the starting point for selecting non-interventional PASS with the following differences:

• Study status: we included only finalised studies since only those would have regulatory outcomes;
• Dates: we considered only studies registered from July 2012 since the PRAC (and respective meeting minutes) were established from that date; and studies finalized by December 2020, allowing an additional 2-year period for studies identified by Sultana et al., to reach completion. For that, another extract of the EU PAS was performed to check the completion status by December 2020 of all studies that were on the original extract.
• MDS definition: the classification of studies as MDS versus non-MDS performed in the study from Sultana et al. was reassessed using a published definition as reference “MDS uses at least two healthcare databases, which are not linked with each other at an individual person level, with analyses carried out in parallel across each database applying a common study protocol” (Gini et al., 2020). Two assessors worked in parallel and blinded to each other. Disagreements were resolved through discussion with a third assessor. After the re-assessment, 151 of the original 319 MDS were retained.
• Study type: we restricted our inclusion to EU safety studies, i.e., PASS, based on inclusion in RMP (category 1, 2, or 3), requested by an EU/UK regulator, and conducted in at least one EU country/UK (i.e., could also include countries from other geographies).

**FIGURE 1:**
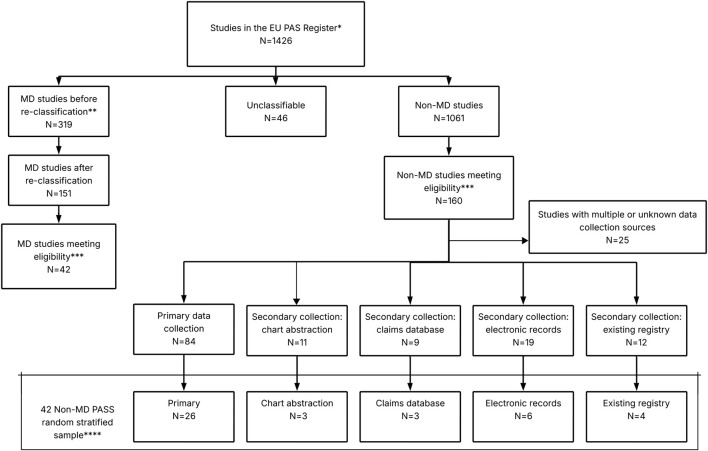
Selection of MDS and non-MDS from EU PAS Register. Abbreviations: MD, Multidatabase. *Extract of studies from Sultana et al. (2022); ** The classification of MD was re-assessed using a published definition as reference: Gini et al. (2020); ***Eligibility criteria: study finalised by December 2020, study requested by an EU/UK regulator, part of the Risk Management Plan (category 1, 2 or 3) and conducted in at least one EU/UK country; ****The proportion of each non-MD PASS data collection type was the same as in the full set of non-MD PASS.

MDS were initially selected applying the above criteria. For non-MDS, the same inclusion criteria were applied and, additionally, studies were stratified by data collection type, using the categories by Sultana et al. (Sultana et al., 2022) Non-MDS whose data collection was classified as “unknown” or “multiple” (i.e., both primary and secondary) were excluded. Finally, a random sample of non-MDS equal to the number of MDS was taken, maintaining the proportion of each non-MDS data collection type the same as in the full set of non-MDS (Figure 1).

### Data extraction for identification and classification of regulatory outcomes

**FIGURE 2:**
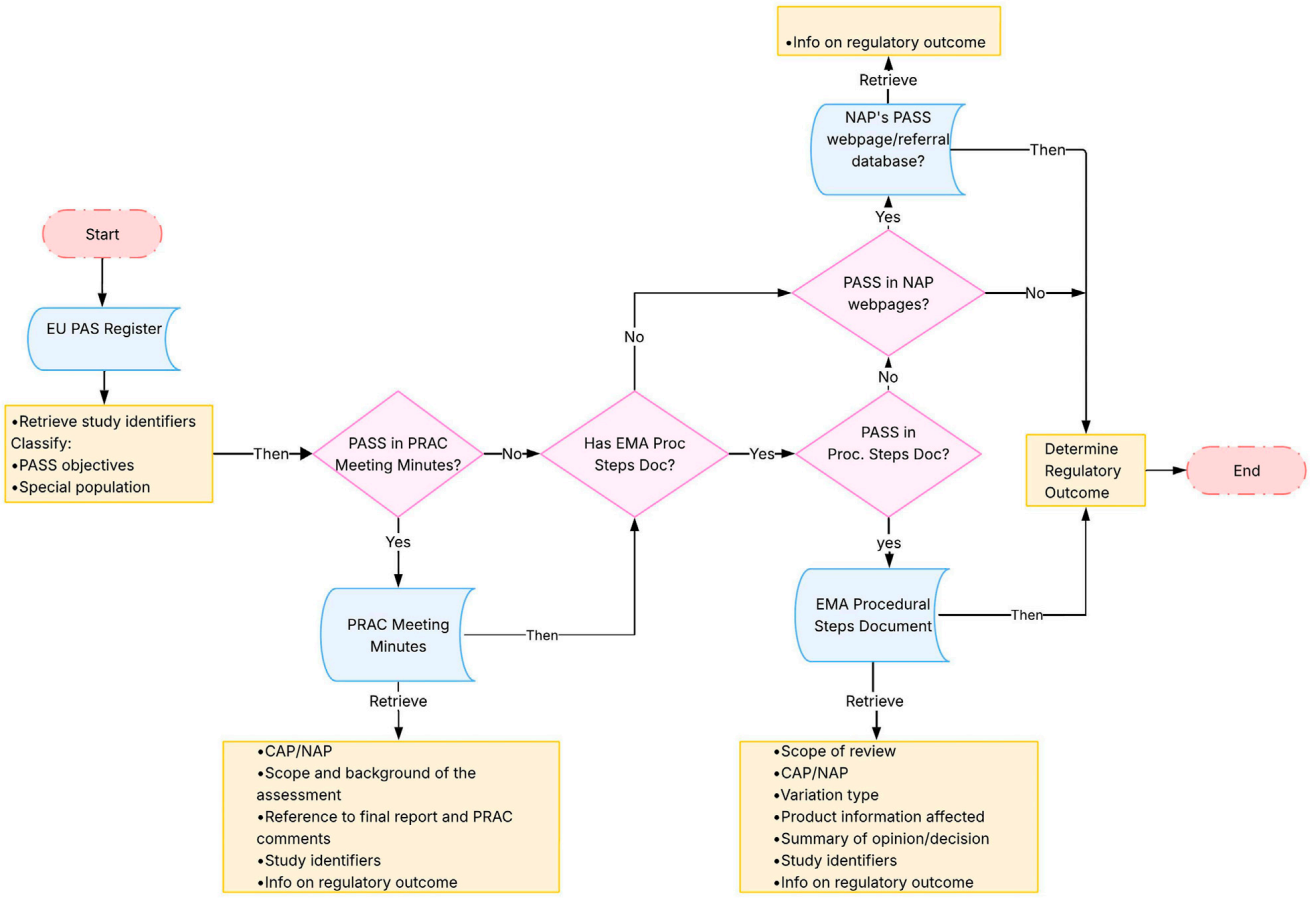
Methodological steps. Abbreviations: CAP, Central Authorisation Procedure; EMA, European Medicines Agency; EU PAS Register, EU electronic Register of Post-Authorisation Studies; NAP, National Authorisation Procedure; PASS, Post-Authorisation Safety Study; PRAC, Pharmacovigilance Risk Assessment Committee; Proc Steps Doc, European public assessment report Procedural Steps Document. Note: PASS were searched in the different documents through study identifiers including study title, acronym or protocol number and regulatory number (product number, agency number or procedure number).

Figure 2 describes how information on regulatory outcomes of the included non-interventional PASS was retrieved from the EMA website. The following steps were applied:

(I) the EU PAS Register^1^ was used to retrieve study identifiers, either directly included in the pre-specified fields (i.e., study title and study acronym) or through reviewing the uploaded documents (i.e., protocol number and regulatory number). Additionally, information on PASS objectives and whether it concerned a special population^2^ was retrieved;
(II) For each included study, the PRAC meeting minutes (European Medicines Agency, 2012), from July 2012 until November 2021 (i.e., covering one additional year from date of study finalisation), were searched using the drug International Nonproprietary Names in combination with the study identifiers found in the EU PAS Register as search terms. The minutes were used to assess the availability of PRAC comments for the PASS and information on regulatory outcomes, and/or to retrieve any regulatory number linked to the final study report assessment, which could facilitate the identification of the regulatory assessments in other sources.
(III) Based on the product name for which a PASS was conducted (when available), the “*Procedural steps taken and scientific information after authorization*” document of the EPAR (European Medicines Agency, 2023) of each product was searched using the regulatory number found in the PRAC minutes, or, if the PASS was not located in the minutes, other available study identifiers from the EU PAS Register. The EPAR Procedural steps document was the main source for retrieving the regulatory outcomes information, as it outlines the regulatory procedures since the authorisation of the medicine (such as variation applications) (European Medicines Agency, 2022) (European Medicines Agency, 2013). The EPAR procedural steps document is available exclusively for CAPs. Regulatory outcome information for PASS involving NAPs was retrieved from the EMA webpage “*Outcomes for active substances contained in nationally authorised products*” (European Medicines Agency, 2023b) and the EMA database of referrals (European Medicines Agency, 2023c).

The studies were evenly divided among eight investigators and information was extracted according to the steps described above, using a common data collection spreadsheet (Supplementary Table 1). Each investigator received a data collection spreadsheet with only the studies assigned to him/her. All extracted information was evaluated independently by another investigator, and discrepancies were discussed in a team meeting until agreement was achieved.

Regulatory outcomes were classified as follows (Table 1): (I) The authors created a predefined list of regulatory outcome labels; (II) one or more outcome labels most closely corresponding to the source text were selected when classifying the studies; (III) Some outcome labels were re-classified: similar labels were collapsed to one outcome and uninformative labels were classified as “unspecified.” These latter labels represented the regulatory outcome types for the purpose of this paper.

**TABLE 1:** Classification of regulatory outcomes.

Predefined regulatory outcome labels selected from source text^a^	Re-classification^b^	Final regulatory outcome classification
Benefit-risk unchanged	—	Benefit-risk unchanged
Change in summary of product characteristics	Change in SmPC and/or PL	Change in SmPC and/or PL
Change in product leaflet		
Implementation of additional pharmacovigilance activities	—	Implementation of additional pharmacovigilance activities
Implementation/reinforcement of a Dear Healthcare Professional Communication (DHPC) or other Risk Minimisation Measures (RMMs)	Implementation of new RMMs or update of RMM	Implementation of new RMMs or update of RMM
Update of RMMs		
Removal from list of additional monitoring	Unspecified	Unspecified
Removal of PASS commitment	Unspecified	Unspecified
Update of Annex II	Unspecified	Unspecified
Update of RMP	Unspecified	Unspecified
Suspension of marketing authorisation	—	Suspension of marketing authorisation
Withdrawal of marketing authorisation	—	Withdrawal of marketing authorisation
Other	—	Other
Not evaluable	—	Not evaluable

^a^ One or more regulatory outcome labels corresponding to the source text could be selected.

^b^ Some regulatory outcome labels were re-classified: some were grouped together for similarity and others were considered “unspecified” as the source text was unspecific and did not inform on the actual regulatory outcome.

The level of confidence in classifying the regulatory outcome(s) for a specific study was considered “certain”, when the available information clearly indicated that the regulatory outcome was a consequence of the concerned PASS (e.g., the EPAR Procedural Steps document mentioned that the SmPC was updated after the submission of the final study report from the particular study), or as “possible,” when the information was unclear (e.g., the regulatory action could have resulted from another PASS or a Periodic Safety Update Report [PSUR] or there was contradictory information between sources). When there was no regulatory outcomes, that information was specifically captured as “missing.”

### Data analysis

The included MDS and non-MDS were analysed descriptively. For the following variables, the number and percentage of studies under each variable category were described: PASS category, scope, involvement of special population, study design, drug type under investigation (biologic or not) and marketing authorisation procedure (CAP or NAP) applicable to the drug. The availability of PASS information and regulatory outcomes across the considered publicly available sources was presented as the number and percentage of MDS/non-MDS PASS. Further stratifications by CAP/NAP were applied. For studies with retrievable regulatory outcomes, the number and percentage were presented for each year of study finalisation and by the MDS/non-MDS classification. In supplementary analyses, the included PASS were stratified by imposed (RMP categories 1 and 2) or non-imposed (RMP category 3).

## Results

### Study description

The study selection process from the EU PAS Register resulted in a total of 84 PASS, 42 MDS and 42 randomly selected non-MDS. Most MDS and non-MDS (∼73%) were non-imposed (Table 2). A higher proportion of MDS (81.0%) than non-MDS (35.7%) investigated drug utilisation, whereas slightly more non-MDS (52.4%) than MDS (42.9%) assessed safety concerns. The marketing authorisation procedure was CAP for half of MDS (54.8%) and most non-MDS (71.4%). The study design and drug types of the studies are described in Supplementary Table 2. Supplementary Table 3 describes the characteristics of the studies stratified by imposed/non-imposed PASS.

**TABLE 2:** The regulatory framework and scope of the included MDS and non-MDS.

	MDS	Non-MDS	Total
	n (%)	n (%)	n (%)
	N = 42	N = 42	N = 84
RMP category			
1 (imposed as condition)	11 (26.2)	11 (26.2)	22 (26.2)
2 (imposed as specific obligation)	1 (2.4)	0 (0.0)	1 (1.2)
3 (non-imposed)	30 (71.4)	31 (73.8)	61 (72.6)
PASS scope			
Drug utilisation	34 (81.0)	15 (35.7)	49 (58.3)
Specific safety concerns	18 (42.9)	22 (52.4)	40 (47.6)
Effectiveness of RMM	12 (28.6)	14 (33.3)	26 (31.0)
Special population			
Yes (e.g., renal impaired, hepatic impaired, immunocompromised, pregnant women, paediatric, elderly)^a^	24 (57.1)	13 (31.0)	37 (44.1)
Marketing authorisation procedure^b^			
CAP	23 (54.8)	30 (71.4)	53 (63.1)
NAP	18 (42.9)	12 (28.6)	30 (35.7)
Mixed^c^	1 (2.4)	0 (0.0)	1 (1.2)

Abbreviations: CAP, Central Authorisation Procedure; MDS, Multidatabase PASS; NAP, National Authorisation Procedure; PASS, Post-Authorisation Safety Study; RMM, Risk Minimisation Measure; RMP, Risk Management Plan.

^a^ Based on EU PAS Register field “Population under study” when there was mention to “other population (e.g., renal impaired, hepatic impaired, immunocompromised, pregnant women) or when there was reference to age <18 years-old and/or >65 years-old if the study description and objective fields in EU PAS Register suggest these age groups were of special interest.

^b^ Referent to the original authorisation procedure by which the drug was approved and not necessarily that the PASS was being conducted to support the initial marketing authorisation procedure.

^c^ Included an active substance for which some brands were approved though central and others through national authorisation procedures.

### PASS traceability across sources and extent of information available

The study protocol and report (or a summary) were uploaded in the EU PAS Register in the majority of cases (>80% of PASS). Of the study identifiers, the EU PAS Register number was mentioned in the regulatory sources for only 5 of the 84 PASS. Thus, other study identifiers were used to track the PASS across the regulatory sources if those identifiers were available in the EU PAS Register.

Overall, 65 of the 84 PASS (77.4%) could be located in at least one of the used sources. From these, 59 PASS, approximately 70% of both MDS and non-MDS, could be retrieved in the PRAC meeting minutes using available study identifiers (Table 3). However, PRAC comments were available for only 16 of them (27.1% of N = 59; corresponding to 19.0% of overall PASS N = 84). The availability of the PASS in the PRAC meeting minutes varied by the drug marketing authorisation procedure: most PASS for CAPs (83.3%) and half of PASS for NAPs (46.7%) were available.^3^ However, PRAC comments were more frequently available for NAPs (36.7%) than CAPs (9.3%). The vast majority of the MDS and non-MDS for CAPs (91.7% and 76.7%, respectively) were found in the EPAR procedural steps document, but less than half had a summary of regulatory comments available (41.7% CAP MDS and 23.3% CAP non-MDS, respectively). Compared to studies for CAPs, MDS and non-MDS for NAPs were less frequently (~43%) located in the sources used as alternative to the EPAR procedural steps document (i.e., NAP/referral outcomes webpages). When stratified by imposed/non-imposed PASS (Supplementary Table 4), imposed PASS (82.6%) were more frequently located in the PRAC meeting minutes than non-imposed (65.6%). Notably, 60.9% of imposed PASS had PRAC comments available compared with only 2 (3.3%) of non-imposed PASS. Non-imposed PASS (62.3%) were more frequently found in the EPAR Procedural Steps document than imposed PASS (30.4%).

**TABLE 3:** PASS traceability across sources and extent of information available (by MDS/non-MDS and CAP/NAP).

Type of information available	MDS			Non-MDS			Total		
	CAP^a^ (N = 24)	NAP (N = 18)	Overall (N = 42)	CAP (N = 30)	NAP (N = 12)	Overall (N = 42)	CAP^a^ (N = 54)	NAP (N = 30)	Overall (N = 84)
Availability of PASS within PRAC minutes, n (%)	20 (83.3)	9 (50.0)	29 (69.0)	25 (83.3)	5 (41.7)	30 (71.4)	45 (83.3)	14 (46.7)	59 (70.2)
PRAC comments available, n (%)	3 (12.5)	6 (33.3)	9 (21.4)	2 (6.7)	5 (41.7)	7 (16.7)	5 (9.3)	11 (36.7)	16 (19.0)
Availability of PASS in Procedural Steps Document (CAP), n (%)	22 (91.7)	NA	22 (52.4)	23 (76.7)	NA	23 (54.8)	45 (83.3)	NA	45 (53.6)
Summary^b^ available, n (%)	10 (41.7)	NA	10 (23.8)	7 (23.3)	NA	7 (16.7)	17 (31.5)	NA	17 (20.2)
Availability in outcomes of NAPs/referral webpages (NAP), n (%)	NA	8 (44.4)	8 (19.0)	NA	5 (41.7)	5 (11.9)	NA	13 (43.3)	13 (15.5)

Abbreviations: CAP, Central Authorisation Procedure; MDS, Multidatabase PASS; NA, Not Applicable; NAP, National Authorisation Procedure; PASS, Post-Authorisation Safety Study; PRAC, Pharmacovigilance Risk Assessment Committee.

^a^ Includes active substances of some brands approved though central and others through national authorisation procedures.

^b^ Summary of the European Public Assessment Report Procedural Steps taken and scientific information after authorisation.

### Availability of regulatory outcomes information

Overall, regulatory outcomes could be retrieved for 39 out of 65 PASS located in at least one of the sources (60.0%; corresponding to 46.4% of all 84 PASS), with an even distribution between MDS and non-MDS and between CAP and NAP (Table 4). Among the PASS with available regulatory outcomes, the regulatory outcome was deemed certainly related to the results of the PASS for 45.0% of the MDS and 73.7% of the non-MDS, and in total for 59.0% of PASS with regulatory outcome available (corresponding to 35.4% of the 65 located PASS and 27.4% of all 84 PASS). When stratifying by authorisation procedure, the regulatory outcome was considered certainly related to the results of the PASS for 80.8% of those for CAP compared to only 15.4% of those for NAP.

**TABLE 4:** Availability of regulatory outcome information.

Availability of Regulatory outcome and level of confidence with classification	MDS			Non-MDS			Total		
	CAP (N = 24)	NAP (N = 18)	Overall (N = 42)	CAP (N = 30)	NAP (N = 12)	Overall (N = 42)	CAP (N = 54)	NAP (N = 30)	Overall (N = 84)
Overall regulatory outcome information available^a^ ^,^ n (%)	12 (50.0)	8 (44.4)	20 (47.6)	14 (46.7)	5 (41.7)	19 (45.2)	26 (48.1)	13 (43.3)	39 (46.4)
Certain, n (% on those available)	9 (75.0)	0 (0.0)	9 (45.0)	12 (85.7)	2 (40.0)	14 (73.7)	21 (80.8)	2 (15.4)	23 (59.0)
Possible, n (% on those available)	3 (25.0)	8 (100.0)	11 (55.0)	2 (14.3)	3 (60.0)	5 (26.3)	5 (19.2)	11 (84.6)	16 (41.0)

Abbreviations: CAP, Central Authorisation Procedure; MDS, Multidatabase PASS; NAP, National Authorisation Procedure.

^a^ Any text related to a regulatory outcome was considered. The level of confidence of an investigator in classifying the regulatory outcome(s) for a specific study was scored as “certain,” whenever the available information clearly indicated that the regulatory outcome was a consequence of the concerned PASS, or “possible” whenever the information was unclear (e.g., the regulatory action could have resulted from another PASS or from a Periodic Safety Update Report [PSUR] or there was contradictory information between sources).

**FIGURE 3:**
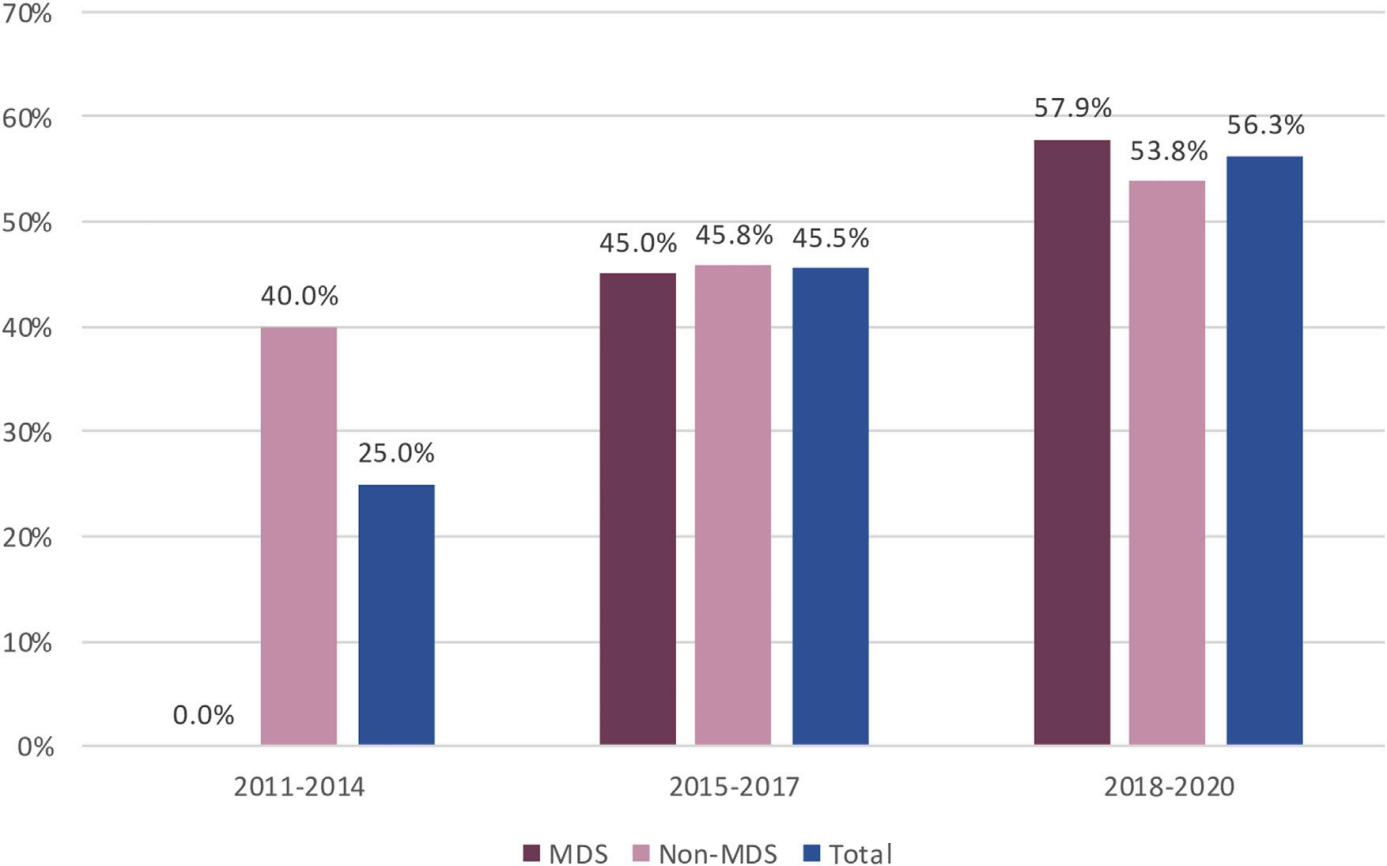
Availability of Regulatory Outcome over time for MDS and non-MDS from EU PAS register. Abbreviations: MDS, Multidatabase PASS.

The availability of regulatory outcomes increased over time, from 25.0% of PASS concluded between 2012 and 2014 to 56.3% of those concluded between 2018 and 2020 (Figure 3). Regulatory outcomes were more frequently available for imposed than non-imposed PASS (78.3% vs. 34.4%, respectively, Supplementary Table 5). While the proportion of PASS with available regulatory outcomes was similar for both marketing authorisation procedures, CAP and NAP (Table 4), the increase over time was only notable for CAP (Supplementary Figure 1).

The frequency of retrieving regulatory outcomes correlated with the presence of the PASS in the PRAC meeting minutes: we retrieved regulatory outcomes for 59.3% of the 59 PASS found the PRAC meeting minutes vs. 16.0% of the 25 PASS not identified in the minutes. Regulatory outcome information was often retrieved from more than one source. Among the 45 PASS with unavailable regulatory outcome information, there was no reference to the final study report and respective outcomes in any of the searched sources (57.8%) or the regulatory outcomes were not evaluable due to lack of information (42.2%) (data not shown).

### Regulatory outcome type

**FIGURE 4:**
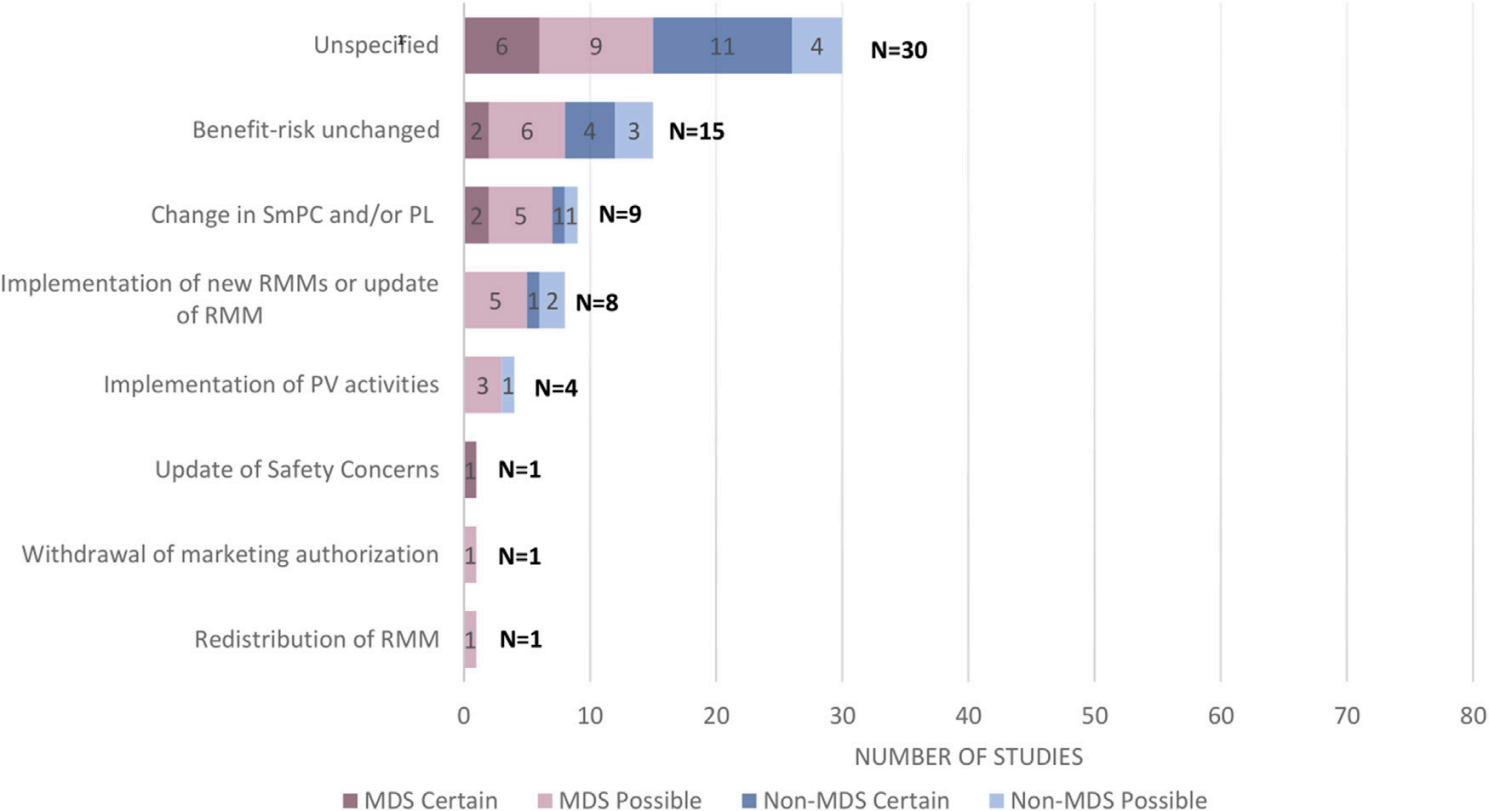
Regulatory outcome types following submission of PASS report (categories are not mutually exclusive) among the 39 PASS with regulatory outcomes. Abbreviations: MDS, Multidatabase PASS; RMM, Risk Minimisation Measure; SmPC, Summary of Product Characteristics; PL, Product Leaflet; PV, Pharmacovigilance. Note 1: “Unspecified” includes: Update of RMP, Update of Annex II, Removal of PASS commitment, Removal from list of additional monitoring. They were reported in 26 PASS (some had more than 1 unspecified outcome). Note 2: The categories are not mutually exclusive and could be overlapping. Note 3: Certain and possible indicate the level of confidence in classifying the regulatory outcome(s) for a specific study.

Among the 39 PASS with available regulatory outcomes, we were able to find 69 regulatory outcomes (on average a PASS had two regulatory outcomes) (Figure 4; Supplementary Figure 2). There were 30 unspecified regulatory outcomes (e.g., “update of RMP,” “removal of PASS commitment”, update of Annex II,” “removal from list of additional monitoring” without any additional information on the nature of the update) mentioned for 26 PASS, which corresponds to two thirds of the PASS with regulatory outcomes. For 15 PASS, the benefit-risk was described as unchanged (for half of them being unclear whether there were some changes to the RMP/product information). The regulatory outcome type was “change in SmPC and/or PL” for 9 PASS and “implementation of new RMM or update of RMM” for 8 PASS. Marketing authorisation withdrawal was reported for one product, although the link to the PASS could not be established with certainty.

## Discussion

This study was the first to identify and describe the regulatory outcomes of PASS from publicly available EMA sources. Overall, 65 of the 84 PASS (77.4%) could be located in at least one of the sources, but only for 39 of those PASS (60%) information on regulatory outcomes was available. The proportion was even lower when considering exclusively regulatory outcomes certainly related to the PASS, i.e. only a third (35.4%) of the 65 located PASS and 27.4% of all 84 PASS had regulatory outcomes certainly related to the PASS. The low number of PASS with available regulatory outcome information limited the ability to draw conclusions on regulatory outcomes by the strata of interest, e.g., MDS vs. non-MDS. The availability of the regulatory outcomes was limited by challenging identification of PASS across the sources, low access to regulatory information and robustness of the regulatory outcome information.

Study identification across the different sources of information that are publicly available on the EMA website was limited by the absence of a unique study identifier (e.g., the EU PAS Register number). To mitigate this limitation, we searched the sources using alternative study identifiers, e.g., protocol number, study acronym, regulatory numbers, and study title. However, study identification remained challenging by the inconsistent and discrepant availability of study identifiers (e.g., inconsistent use of acronyms, protocol numbers, product numbers, and procedure numbers) across sources. Moreover, locating identifiers relied on documents being uploaded in the EU PAS Register. Consistent with previous publications (Engel and Almas et al., 2017; Sultana et al., 2022; Jouaville et al., 2021), protocols and reports are not uploaded for all studies in the EU PAS Register, despite explicit recommendation in the GVP (European Medicines Agency, 2017) and ENCePP Code of Conduct. (The European Network of Centres for Pharmacoepidemiology and Pharmacovigilance, 2018). However, we observed a higher proportion of studies with available protocol (>80%) than previously reported (ca 60%) (Sultana et al., 2022), potentially due to our focus on studies required by regulators, where greater transparency is expected. Nonetheless, our findings further reinforce the importance of improving study registration and document deposition, as emphasised previously. (Gini et al., 2019). The same recommendation resulted from a study examining post marketing studies registration, results reporting and publication in the United States (US), which found that only approximately three quarters of the postmarketing requirements were registered on ClinicalTrials.gov, and nearly three quarters of completed studies reported results or were published. (Wallach et al., 2018).

The identification of the PASS in the PRAC meeting minutes facilitated locating the PASS in the Procedural steps document or the sources used for NAPs, as the full/partial reference number in the PRAC meeting minutes was typically used in the other documents. However, it was complicated to locate PASS in the PRAC meeting minutes, as previously acknowledged (Engel and Almas et al., 2017), and we could locate only around two thirds of the PASS in the minutes even when reviewing up to an additional year after the date of finalisation of the most recent PASS. Imposed PASS were more frequently identified in the PRAC meeting minutes and had more PRAC comments available compared to non-imposed, likely because the PRAC plenary meetings tend to focus on imposed PASS. The frequency of locating PASS across sources did not differ between MDS and non-MDS. The above-mentioned US study found similar methodological challenges and recommended the addition of ClinicalTrials.gov National Clinical Trial (NCT) numbers to postmarketing requirement descriptions and the postmarketing study and clinical trial requirements and commitments database files to facilitate linking study listings to FDA documents for postmarketing studies. (Wallach et al., 2018).

Regulatory outcomes were identified for fewer PASS than could be located in at least one of the sources due to lack of information. For example, PRAC comments were typically unavailable and only a minority of the identified EPAR procedural steps documents included regulatory outcome information. The availability of regulatory outcome information was consistently low between MDS and non-MDS.

Concerning trends between imposed and non-imposed PASS, regulatory outcomes were expectedly more frequently available for imposed PASS. However, our finding that regulatory outcomes were not available for all imposed PASS was unexpected, as we *a priori* anticipated their availability according to the EMA website. (European Medicines Agency, 2023b). All imposed PASS without available regulatory outcomes were for NAPs (2 of the 5 in the UK), which likely explains the absence of information on the EMA website. However, our results should be interpreted considering a possible misclassification of imposed/non-imposed PASS, because the classification was self-registered in the EU PAS Register. As highlighted previously, the self-registration lacks systematic external quality control or validation. (Sultana et al., 2022).

While regulatory outcomes were available equally rarely for PASS for NAPs and CAPs, the type of documents for NAPs and CAPs differed in structure and content and thereby the type of information available. For CAPs, whenever the PASS was mentioned in the document “Procedural steps taken and scientific information after authorization” it was identified as a dedicated item within the document making it clear the conclusion was specific to the study. However, for NAPs, most of the retrieved documents were conclusions to referral procedures where the PASS was among several other investigations that were done and discussed altogether. Therefore, the relationship between regulatory outcomes and a specific PASS could not be established with certainty, as the documents discussed regulatory outcomes resulting from several pharmacovigilance activities. In addition, for both CAPs and NAPs, the available regulatory outcome for a specific PASS was considered only “possibly” related to the PASS, when information between different sources conflicted (e.g., PRAC meeting minutes and EPAR procedural steps document). Thus, overall, we were uncertain of the relationship between the regulatory outcome and the specific PASS for 41% of the PASS with available outcome information, either because the source(s) referred to several studies or processes, or information between sources conflicted (i.e., classified as “possible” regulatory outcomes).

Time between study conclusion and publication of regulatory outcomes do not seem to explain the limited regulatory outcome publication, as the most recently finalised studies tended to have more regulatory outcomes available. In fact, the results, even if limited by the low numbers, suggest an increase in the availability of the regulatory outcome information of PASS for CAPs over time, which reflects an overall increase in regulatory outcome information for the last 3 years covered in the analysis.

Even when regulatory outcome information was available, the quality of the data precluded an informative description of the regulatory outcomes, such as, the lack of granularity, standardisation and consistency across sources of information. Thus, the regulatory outcome type was frequently categorised as unspecified. For example, the most common text found was “update of RMP,” without specifying the nature of the update.

With the increased use of post-marketing studies for medicinal product benefit-risk assessment, it will be important to strengthen study registration, results reporting and publication of regulatory outcomes to promote transparency and ensure the translation of the study results into clinical practice. (Wallach et al., 2018).

While we attempted to identify PASS regulatory outcomes from publicly accessible sources, we acknowledge that these sources were not originally intended for this purpose. The use of a subset of studies re-classified as MDS by using a common definition is a strength of our study, even though it resulted in a lower number of studies. Another strength of this study was the execution of a quality control check of the dataset, where all entries and variables were reviewed by two investigators among those involved in the data extraction, allowing for further alignment in classification. Any discrepancies were discussed and resolved in team meetings.

In addition to the challenges in classifying the regulatory outcomes of studies, we also encountered limitations regarding the level and accuracy of information and availability of documents (e.g., deposited protocol) reported in the EU PAS Register, as pointed out by Sultana et al. (2022). The EU PAS Register has recently been replaced by the “HMA-EMA Catalogue of real-world studies,” which is expected to ease its use and ideally improve the quality and completeness of registered information (EMA, 2025). To increase the upload of study documents such as protocols and reports, we would recommend strengthening the guidance and implement a tool that would require applicants to upload the protocol whenever they want to update the study information shown on the Catalogue.

In conclusion, the attempt to identify and describe the regulatory outcomes of MDS registered into the EU PAS Register, and compare them to non-MDS, was impaired by the difficulties in retrieving the regulatory outcome insights from the publicly available sources. Although 77% of the PASS could be located in at least one of the sources, information on regulatory outcomes was only available for 60% of those PASS (and 46% of the overall 84 PASS). The availability of the regulatory outcomes was limited by challenges in identifying PASS across the sources and lack of information on regulatory outcomes. Even when regulatory outcome information was available, the quality of the data precluded an informative description of the regulatory outcomes, due to the lack of granularity, standardisation and consistency across sources of information. Therefore, we advocate for increasing transparency of the regulatory outcomes of PASS to further translate study results into clinical practice. Our recommendations which emerged from our results are: (i) to implement a consistent, unique study identifier used across all sources for PASS, from initial PASS request to start of the PASS and then throughout all subsequent stages to allow identification of PASS information; (ii) to publish regulatory outcomes information for all PASS, and in particularly for NAPs; and (iii) to standardise the presentation of regulatory outcomes information with sufficient detail to understand the regulatory impact of the PASS.

## Prior postings and presentations

The work described in this publication was presented as a spotlight poster presentation at ICPE 2024 (Publication number 1170).

## Data Availability

The raw data supporting the conclusion of this article will be made available by the authors, without undue reservation.

## Footnotes

1 Recently, the HMA-EMA Catalogues of real-world data sources and studies has replaced the EU PAS Register and the ENCePP Resource Database.

2 Based on EUPAS Register field “Population under study” when there was mention to “other population (e.g., renal impaired, hepatic impaired, immunocompromised, pregnant women) or, when there was reference to age <18 years-old and/or >65 years-old if the study description and objective fields in EU PAS Register suggest these age groups were of special interest.

3 To note that this refers to the original authorisation procedure by which the drug was approved and not necessarily that the PASS was being conducted to support the initial marketing authorisation procedure.
