# Supplementary figures and images for: Identifying regulatory outcomes of Non-interventional Post-Authorisation Safety Studies (PASS) in the European repository of studies using publicly available information
Source: Front Drug Saf Regul. 2025 Sep 10;5:1574430. doi: 10.3389/fdsfr.2025.1574430 (PMC12443101; doi:10.3389/fdsfr.2025.1574430)

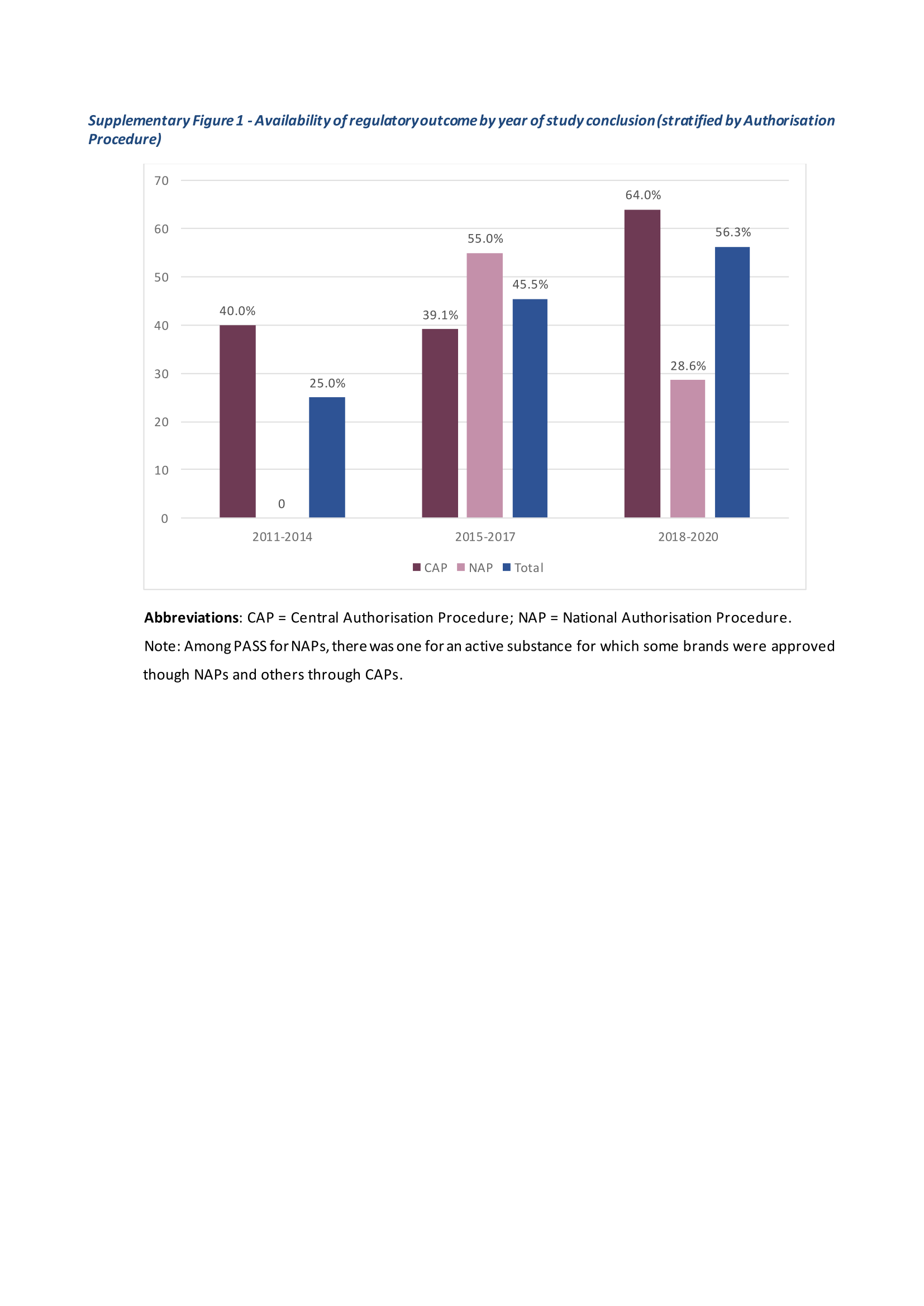

Supplement: Supplementary file 1 [file Image1.tiff]

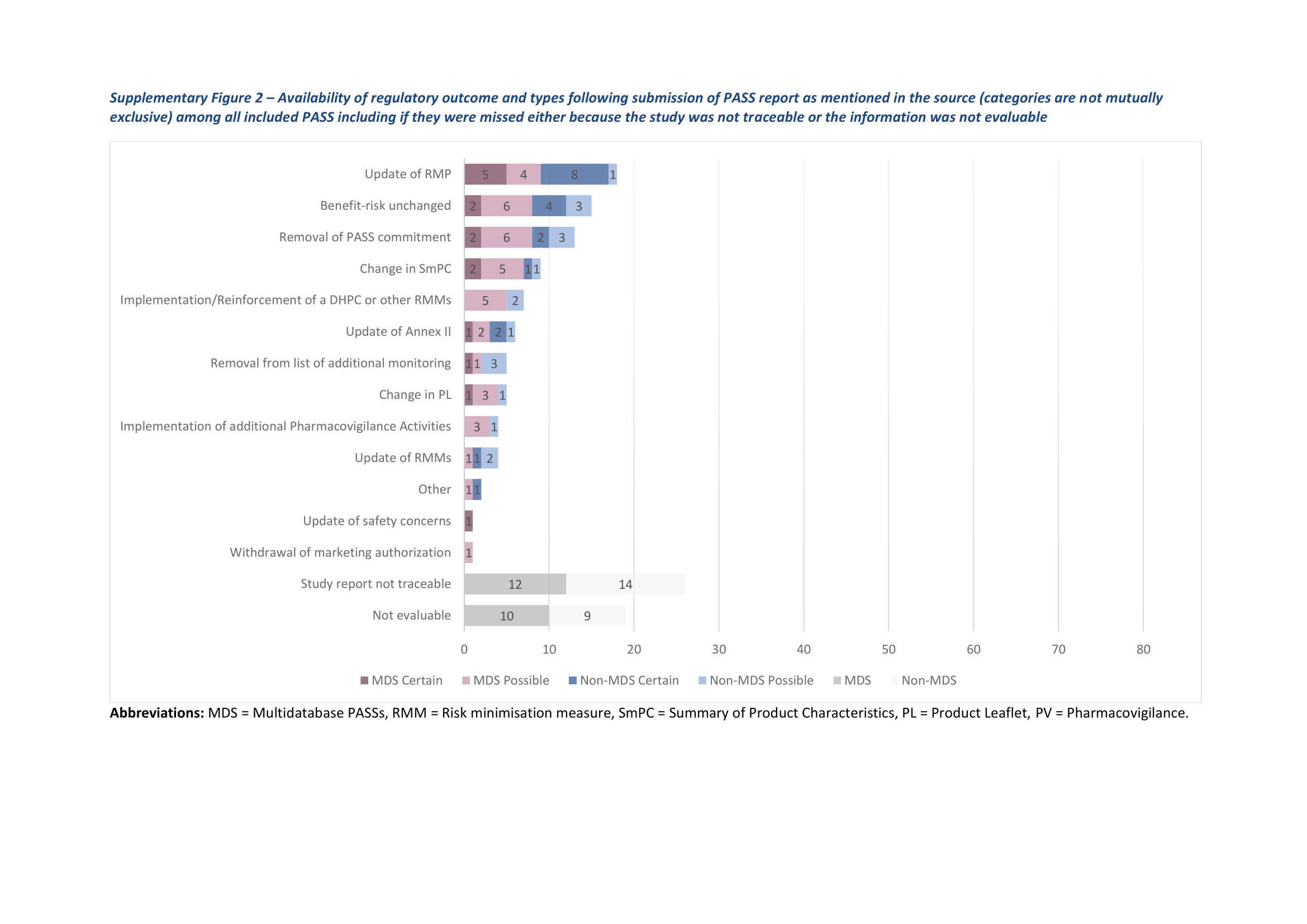

Supplement: Supplementary file 7 [file Image2.tiff]
