# Supplementary material for: Identifying regulatory outcomes of Non-interventional Post-Authorisation Safety Studies (PASS) in the European repository of studies using publicly available information
Source: Front Drug Saf Regul. 2025 Sep 10;5:1574430. doi: 10.3389/fdsfr.2025.1574430 (PMC12443101; doi:10.3389/fdsfr.2025.1574430)
Supplement: Supplementary file 2 [file Table1.docx]

# Supplementary Material

***Supplementary Table 1 – Data captured in the data collection spreadsheet***

| **Data element** | **Description (indication if copied from source or derived)** | **Source** | **Categories** |
| --- | --- | --- | --- |
| EU PAS Register number | Copied | EU PAS Register | Not applicable |
| MDS/Non-MDS | Copied | Sultana et al dataset^1^ (with additional validation on MDS definition) | MDS |
|  |  |  | Non-MDS (Primary data collection; Secondary data collection [chart review]; Secondary data collection [claims database]; Secondary data collection [electronic health record]; Secondary data collection [existing registry]) |
| Date of Final Study Report | Copied | EU PAS Register | 2012-2014; 2015-2017; 2018-2020 |
| Study title | Copied | EU PAS Register | Not applicable |
| Study type | Copied | EU PAS Register | Active surveillance; Observational Study; Other |
| Brief Description of the Study | Copied | EU PAS Register | Not applicable |
| Is the study required by a Risk Management Plan (RMP)? | Copied | EU PAS Register | EU RMP category 1 (imposed as condition of marketing authorisation); EU RMP category 2 (specific obligation of marketing authorisation); EU RMP category 3 (required) |
| Countries in which this study is being conducted | Copied | EU PAS Register | Not applicable |
| Is this study being carried out with an established data source? | Copied | EU PAS Register | yes; no |
| Sources of data | Copied | EU PAS Register | Not applicable |
| What is the scope of the study? | Copied | EU PAS Register | Not applicable |
| Primary scope | Copied | EU PAS Register | Not applicable |
| What is the main objective of the study? | Copied | EU PAS Register | Not applicable |
| Full protocol available | Derived | EU PAS Register | Document; not submitted |
| Study Results available | Derived | EU PAS Register | Document; not submitted |
| Other study registration identification numbers and URLs as applicable | Copied | EU PAS Register | Not applicable |
| Study drug information: Brand product name | Copied | EU PAS Register | Not applicable |
| Study drug information: active substance (INN) | Copied | EU PAS Register | Not applicable |
| Study drug information: substance class (ATC) | Copied | EU PAS Register | Not applicable |
| Study design | Copied | Sultana et al dataset^1^ | Study design |
| Use of reference drug for formal comparison | Copied | Sultana et al dataset^1^ | Use of reference drug for formal comparison |
| Drug type | Copied | Sultana et al dataset^1^ | Drug type |
| Marketing authorisation procedure | Derived (taken from PRAC meeting minutes for those studies found there; otherwise, public data from article 57 database was consulted by product name (if available) or active substance – if unclear (e.g. marketing authorisation may have changed over time) – the EMA’s medicine-related data spreadsheet available on the website was used as a complement | PRAC meeting minutes, public data from article 57 database^2^ and EMA’s medicine-related data spreadsheet^3^ | Marketing authorisation procedure |
| Availability of study acronym in EU PAS Register | Derived | EU PAS Register (including uploaded documents if available) | yes; no |
| Study acronym | Copied | EU PAS Register (including uploaded documents if available) | Not applicable |
| Availability of protocol number in EU PAS Register | Derived | EU PAS Register (including uploaded documents if available) | yes; no |
| Protocol number | Copied | EU PAS Register (including uploaded documents if available) | Not applicable |
| Availability of EMA regulatory number | Derived | EU PAS Register (including uploaded documents if available) | yes; no |
| EMA regulatory number | Copied (if more than one number was available, all were considered) | EU PAS Register (including uploaded documents if available) | Not applicable |
| PASS Scope: Assess safety concerns | Derived (study objectives mentions focus on assessing any safety concerns) | EU PAS Register (including uploaded documents if available) | yes; no |
| PASS scope: Drug Utilisation | Derived (study objectives mentions focus on drug utilisation, drug use patterns including off-label use) | EU PAS Register (including uploaded documents if available) | yes; no |
| PASS scope: assesses effectiveness of RMM | Derived (study objectives mentions the assessment of the effectiveness of risk minimisation measures e.g., educational materials, pregnancy prevention programme, etc.) | EU PAS Register (including uploaded documents if available) | yes; no |
| PASS scope: Special Population | Derived (if EU PAS Register field “Population under study” mentioned only age and gender consider “no” unless there is an age group <18 years-old or >65-years old, in which case, we considered also the fields “Brief description of the study” and “Main objective(s)” and if those age groups were also specifically mentioned, we considered it was a special population. In addition, if an additional category was mentioned under “other population” (e.g renal impaired), we considered it a special population) | EU PAS Register (including uploaded documents if available) | yes; no |
| PASS found in PRAC meeting minutes | Derived (searching for PASS using any of the available study identifiers) | PRAC meeting minutes | yes; no |
| PRAC meeting minutes: Year and Month the meeting occurred | Copied | PRAC meeting minutes | Not applicable |
| PRAC meeting minutes: minutes' PASS related sub-section in which study is found | Copied | PRAC meeting minutes | Protocol/ Results/ Blank if not classifiable) |
| PRAC meeting minutes: EMA Procedure Number | Copied | PRAC meeting minutes | Not applicable |
| PRAC meeting minutes: Entry related to PASS mentioned study acronym? | Derived | PRAC meeting minutes | yes; no |
| PRAC meeting minutes: Entry related to PASS mentioned protocol number? | Derived | PRAC meeting minutes | yes; no |
| PRAC meeting minutes: Entry related to PASS mentioned study title? | Derived | PRAC meeting minutes | yes; no |
| PRAC meeting minutes: Procedure Scope | Copied | PRAC meeting minutes | Not applicable |
| PRAC meeting minutes: Background information | Copied | PRAC meeting minutes | Not applicable |
| PRAC meeting minutes: PRAC comments | Copied | PRAC meeting minutes | Not applicable |
| PRAC meeting minutes: Is there an entry related with the final study report? | Derived | PRAC meeting minutes | yes; no |
| PASS found in EPAR Procedural Steps? | Derived (searching for PASS using any of the available study identifiers) | EPAR of the concerned medicinal product covered in the PASS– Procedural steps taken and scientific information after authorisation | yes; no; Not applicable (e.g. PASS concerns a NAP) |
| EPAR Procedural Steps: Entry related to PASS mentioned study acronym? | Derived | EPAR – Procedural steps taken and scientific information after authorisation | yes; no |
| EPAR Procedural Steps: Entry related to PASS mentioned protocol number? | Derived | EPAR – Procedural steps taken and scientific information after authorisation | yes; no |
| EPAR Procedural Steps: Entry related to PASS mentioned study title? | Derived | EPAR – Procedural steps taken and scientific information after authorisation | yes; no |
| EPAR Procedural Steps: Application Number | Copied | EPAR – Procedural steps taken and scientific information after authorisation | Not applicable |
| EPAR Procedural Steps: Scope | Copied | EPAR – Procedural steps taken and scientific information after authorisation | Not applicable |
| EPAR Procedural Steps: Variation code(s) | Copied | EPAR – Procedural steps taken and scientific information after authorisation | Not applicable |
| EPAR Procedural Steps: Opinion/Notification issued on; Commission Decision Issued / amended on | Copied (if both available, took the Commission Decision date) | EPAR – Procedural steps taken and scientific information after authorisation | Date |
| EPAR Procedural Steps: Product Information affected | Copied | EPAR – Procedural steps taken and scientific information after authorisation | SmPC; PL; Annex II |
| EPAR Procedural Steps: Summary | Copied | EPAR – Procedural steps taken and scientific information after authorisation | Not applicable |
| PASS results available in other source? (for PASS involving NAP) | Derived | EMA website: “Outcomes for active substances contained in nationally authorised products” (10) and the EMA database of referrals (REF). | yes; no |
| Reg Outcome info available? | Derived | Based on all information retrieved in the abovementioned documents | yes; no |
| Reg Outcomes as provided in the source | Derived | Based on all information retrieved in the abovementioned documents | See predefined regulatory outcome labels selected from source text in Table 1 |
| Reg Outcomes as (re-classified) | Derived | Based on “Regulatory Outcomes as provided in the source” | See final regulatory outcome classification in Table 1 |
| Ascertainment level | Derived based on criteria described in Methods | Based on all information retrieved in the abovementioned documents | Certain; Possible |
| Reason for “possible” | Derived based on criteria described in Methods | Based on all information retrieved in the abovementioned documents | Not applicable |
| Supporting evidence for Reg Outcome | Derived (documents where regulatory outcome information was found) | EU PAS Register; PRAC Meeting Minutes; EPAR Procedural Steps; Other[specify] | EU PAS Register; PRAC meeting minutes; EPAR Procedural; Other[specify] |

*1 Sultana J, Crisafulli S, Almas M, Antonazzo IC, Baan E, Bartolini C, et al. Overview of the European post‐authorisation study register post‐authorization studies performed in Europe from September 2010 to December 2018. Pharmacoepidemiology and Drug Safety. 2022;31(6):689-705*

*^2^ European Medicines Agency. Public data from Article 57 database [available from* [*https://www.ema.europa.eu/en/human-regulatory-overview/post-authorisation/data-medicines-iso-idmp-standards-post-authorisation/public-data-article-57-database*](https://www.ema.europa.eu/en/human-regulatory-overview/post-authorisation/data-medicines-iso-idmp-standards-post-authorisation/public-data-article-57-database)*]*

*^3^ European Medicines Agency. Download medicine data [available from* *https://www.ema.europa.eu/en/medicines/download-medicine-data]*
