# Supplementary material for: Identifying regulatory outcomes of Non-interventional Post-Authorisation Safety Studies (PASS) in the European repository of studies using publicly available information
Source: Front Drug Saf Regul. 2025 Sep 10;5:1574430. doi: 10.3389/fdsfr.2025.1574430 (PMC12443101; doi:10.3389/fdsfr.2025.1574430)
Supplement: Supplementary file 3 [file Table2.docx]

# Supplementary Material

***Supplementary Table 2 - MDS and non-MDS PASS design and drug type***

|  | **MDS** | **Non-MDS** | **Total** |
| --- | --- | --- | --- |
|  | **n (%)** | **n (%)** | **n (%)** |
|  | **N= 42 (100)** | **N= 42 (100)** | **N= 84 (100)** |
| **Study design** | | | |
| **Cohort study** | 17 (40.5) | 12 (28.6) | 29 (34.5) |
| **Descriptive study** | 13 (31.0) | 16 (38.1) | 29 (34.5) |
| **Cross-sectional study** | 6 (14.3) | 7 (16.7) | 13 (15.5) |
| **Nested case-control study** | 2 (4.8) | 2 (4.8) | 4 (4.8) |
| **More than 1 study design** | 3 (7.1) | 1 (2.4) | 4 (4.8) |
| **Unknown** | 0 (0.0) | 3 (7.1) | 3 (3.6) |
| **Other types of analytic studies** | 0 (0) | 1 (2.4) | 1 (1.2) |
| **Case-control study** | 1 (2.4) | 0 (0.0) | 1 (1.2) |
| **Inclusion of a Comparison Group** | | | |
| **Yes** | 15 (35.7) | 6 (14.3) | 21 (25.0) |
| **No** | 26 (61.9) | 34 (81.0) | 60 (71.4) |
| **Unknown** | 1 (2.4) | 2 (4.8) | 3 (3.6) |
| **Drug type** | | | |
| **Biologic** | 3 (7.1) | 8 (19.0) | 11 (13.1) |
| **Non-biologic** | 35 (83.3) | 32 (76.2) | 67 (79.8) |
| **None** | 1 (2.4) | 1 (2.4) | 2 (2.4) |
| **Unknown** | 3 (7.1) | 1 (2.4) | 4 (4.8) |

**Abbreviations**: MDS = Multidatabase PASS; RMM = Risk Minimisation Measure.

Variables’ categories were taken from the dataset of *Sultana J, Crisafulli S, Almas M, Antonazzo IC, Baan E, Bartolini C, et al. Overview of the European post‐authorisation study register post‐authorization studies performed in Europe from September 2010 to December 2018. Pharmacoepidemiology and Drug Safety. 2022;31(6):689-705*. See Supplementary Material Table 1.
