# Supplementary material for: Identifying regulatory outcomes of Non-interventional Post-Authorisation Safety Studies (PASS) in the European repository of studies using publicly available information
Source: Front Drug Saf Regul. 2025 Sep 10;5:1574430. doi: 10.3389/fdsfr.2025.1574430 (PMC12443101; doi:10.3389/fdsfr.2025.1574430)
Supplement: Supplementary file 4 [file Table5.docx]

# Supplementary Material

***Supplementary Table 5 – Availability of regulatory outcome information (by imposed/non-imposed PASS)***

|  | **Imposed (RMP Category 1 and 2)^a^** | **Non-imposed (RMP Category 3)** | **Total** |
| --- | --- | --- | --- |
|  | **N=23** | **N=61** | **N=84** |
| **Overall regulatory Outcome information available^1, n(%)^** | **18 (78.3)** | **21 (34.4)** | **39 (46.4)** |
| **Certain, n(% on those available)** | 7 (38.9) | 16 (76.2) | 23 (59.0) |
| **Possible, n(% on those available)** | 11 (61.1) | 5 (23.8) | 16 (41.0) |

**Abbreviations**: RMP = Risk Management Plan

^1^ Any text related to a regulatory outcome was considered. The level of confidence of an investigator in classifying the regulatory outcome(s) for a specific study was also scored as “certain”, whenever the available information clearly indicate that the regulatory outcome was a consequence of the concerned PASS, or “possible” whenever the information was unclear (e.g., the regulatory action could have resulted from another PASS or from a Periodic Safety Update Report [PSUR] or there was contradictory information between sources).

^a^ There was only one PASS of RMP category 2
