# Supplementary material for: Identifying regulatory outcomes of Non-interventional Post-Authorisation Safety Studies (PASS) in the European repository of studies using publicly available information
Source: Front Drug Saf Regul. 2025 Sep 10;5:1574430. doi: 10.3389/fdsfr.2025.1574430 (PMC12443101; doi:10.3389/fdsfr.2025.1574430)
Supplement: Supplementary file 5 [file Table3.docx]

# Supplementary Material

***Supplementary Table 3- Description of studies by PASS RMP category***

|  | **Imposed^1^** | **Non-imposed** | **Total** |
| --- | --- | --- | --- |
|  | **n (%)** | **n (%)** | **n (%)** |
|  | **N= 23** | **N= 61** | **N= 84** |
| **MDS/Non-MDS** | | | |
| **MDS** | 12 (52.2) | 30 (49.2) | 42 (50.0) |
| **Non-MDS** | 11 (47.8) | 31 (50.8) | 42 (50.0) |
| **PASS scope** | | | |
| **Drug utilisation** | 16 (69.6) | 33 (54.1) | 49 (58.3) |
| **Assess safety concerns** | 11 (47.8) | 29 (47.5) | 40 (47.6) |
| **Assess effectiveness of RMM** | 11 (47.8) | 15 (24.6) | 26 (31.0) |
| **Special Population** | | | |
| **Yes (**e.g renal impaired, hepatic impaired, immunocompromised, pregnant women, paediatric, elderly)^2^ | 7 (30.4) | 30 (49.2) | 37 (44.0) |
| **Study design** | | | |
| **Cohort study** | 8 (34.8) | 21 (34.4) | 29 (34.5) |
| **Descriptive study** | 8 (34.8) | 21 (34.4) | 29 (34.5) |
| **Cross-sectional study** | 6 (26.1) | 7 (11.5) | 13 (15.5) |
| **Nested case-control study** | 0 (0.0) | 4 (6.6) | 4 (4.8) |
| **More than 1 study design** | 1 (4.3) | 3 (4.9) | 4 (4.8) |
| **Unknown** | 0 (0.0) | 3 (4.9) | 3 (3.6) |
| **Other types of analytic studies** | 0 (0) | 1 (1.6) | 1 (1.2) |
| **Case-control study** | 0 (0) | 1 (1.6) | 1 (1.2) |
| **Comparative** | | | |
| **Yes** | 4 (17.4) | 17 (27.9) | 21 (25.0) |
| **No** | 18 (78.3) | 42 (68.9) | 60 (71.4) |
| **Unknown** | 1 (4.3) | 2 (3.3) | 3 (3.6) |
| **Drug type** | | | |
| **Biologic** | 0 (0) | 11 (18.0) | 11 (13.1) |
| **Non-biologic** | 20 (87) | 47 (77.1) | 67 (79.8) |
| **None** | 0 (0) | 2 (3.3) | 2 (2.4) |
| **Unknown** | 3 (13.0) | 1 (1.6) | 4 (4.8) |
| **Marketing authorisation procedure^3^** | | | |
| **CAP** | 7 (30.4) | 46 (75.4) | 53 (63.1) |
| **NAP** | 16 (69.6) | 14 (23.0) | 30 (35.7) |
| **Mixed^4^** | 0 (0.0) | 1 (1.6) | 1 (1.2) |

**Abbreviations**: CAP = Central Authorisation Procedure; MDS = Multidatabase PASS; NAP = National Authorisation Procedure; RMM = Risk Minimisation Measure; RMP = Risk Management Plan.

Variables’ categories were taken from the dataset of Sultana J, Crisafulli S, Almas M, Antonazzo IC, Baan E, Bartolini C, et al. Overview of the European post‐authorisation study register post‐authorization studies performed in Europe from September 2010 to December 2018.

^1^ There was only one PASS of RMP category 2

**^2^** Based on EU PAS Register field “Population under study” when there was mention to “other population (e.g renal impaired, hepatic impaired, immunocompromised, pregnant women) or when there was reference to age <18 years-old and/or >65 years-old if the study description and objective fields in EU PAS Register suggest these age groups were of special interest.

^3^ Referent to the original authorisation procedure by which the drug was approved and not necessarily that the PASS was being conducted to support the initial marketing authorisation procedure.

^4^ Included an active substance for which some brands were approved though central and others through national authorisation procedures.
