# Supplementary material for: Identifying regulatory outcomes of Non-interventional Post-Authorisation Safety Studies (PASS) in the European repository of studies using publicly available information
Source: Front Drug Saf Regul. 2025 Sep 10;5:1574430. doi: 10.3389/fdsfr.2025.1574430 (PMC12443101; doi:10.3389/fdsfr.2025.1574430)
Supplement: Supplementary file 6 [file Table4.docx]

# Supplementary Material

***Supplementary Table 4 - PASS traceability across sources and extent of information available (by imposed/non-imposed PASS)***

|  | **Imposed (RMP Category 1 and 2)^a^** | | | **Non-imposed (RMP Category 3)** | | | **Total** | | |
| --- | --- | --- | --- | --- | --- | --- | --- | --- | --- |
|  | CAP (N=7) | NAP  (N=16) | Overall  (N=23) | CAP^b^  (N=47) | NAP  (N=14) | Overall  (N=61) | CAP^b^  (N=54) | NAP  (N=30) | Overall  (N=84) |
| **Availability of PASS within PRAC minutes, n(%)** | 7 (100.0) | 12 (75.0) | 19 (82.6) | 38 (80.9) | 2 (14.3) | 40(65.6) | 45 (83.3) | 14 (46.7) | 59 (70.2) |
| **PRAC comments available, n(%)** | 3 (42.9) | 11 (68.8) | 14 (60.9) | 2 (4.3) | 0 (0.0) | 2 (3.3) | 5 (9.3) | 11 (36.7) | 16 (19.0) |
| **Availability of PASS in Procedural Steps Document (CAP), n(%)** | 7 (100) | NA | 7 (30.4) | 38 (80.9) | NA | 38 (62.3) | 45 (83.3) | NA | 45 (53.6) |
| **Summary^c^ available, n(%)** | 6 (85.7) | NA | 6 (26.1) | 11 (23.4) | NA | 11 (18.0) | 17 (31.5) | NA | 17 (20.2) |
| **Availability in outcomes of NAPs/referral webpages (NAP), n(%)** | NA | 11 (68.8) | 11 (47.8) | NA | 2 (14.3) | 2 (3.3) | NA | 13 (43.3) | 13 (15.5) |

**Abbreviations**: CAP = Central Authorisation Procedure; NA = Not Applicable; NAP = National Authorisation Procedure; PASS = Post-Authorisation Safety Study; PRAC = Pharmacovigilance Risk Assessment Committee; RMP = Risk Management Plan.

^a^ There was only one PASS of RMP category 2

^b^ Includes an active substance that had some brands approved though central and others through national authorisation procedures

^c^ Summary of the European Public Assessment Report Procedural Steps taken and scientific information after authorisation
